# Phytochemical Composition and Chronic Hypoglycemic Effect of* Bromelia karatas* on STZ-NA-Induced Diabetic Rats

**DOI:** 10.1155/2019/9276953

**Published:** 2019-02-25

**Authors:** Sonia M. Escandón-Rivera, Adolfo Andrade-Cetto, Gabriela Sánchez-Villaseñor

**Affiliations:** Laboratorio de Etnofarmacología, Facultad de Ciencias, Universidad Nacional Autónoma de México, 4510 Ciudad de México, Mexico

## Abstract

Oral administration of an aqueous extract of the aerial parts of* Bromelia karatas* to STZ-NA rats showed a significant hypoglycemic effect in a chronic trial lasting 42 days. Chromatographic profiles of the active extract (WE) and an organic extract (OE) of* B. karatas* were obtained by high-performance liquid chromatography (HPLC) and used to identify their major components. Isolation and identification of the compounds present in the extracts were accomplished by means of various conventional chromatographic and spectroscopic techniques. This process led to the identification of *β*-sitosterol-3-O-*β*-D-glucopyranoside (**1**) and *ρ*-coumaric acid (**3**) as the major compounds present in the extracts. During the isolation of** 1** and** 3**, seven additional metabolites not previously reported for the plant were obtained, namely, cirsiliol 4′-O-*β*-D-glucopyranoside (**2**), stigmasterol (**4**), *β*-sitosterol (**5**), 1-O-feruloyl-3-O-*ρ*-coumaroylglycerol (**6**), *β*-D-(1-O-acetyl-3,6-O-trans-diferuloyl) fructofuranosyl-*α*-D-2′,4′,6′-O-triacetylglucopyranoside (**7**), 1-O-p-coumaroyl-3-O-caffeoylglycerol (**8**), and 2-propyl-*β*-glucopyranoside (**9**).

## 1. Introduction

Diabetes mellitus is a metabolic disease that is characterized by elevated levels of blood glucose that over time cause serious damage to the heart, blood vessels, eyes, kidneys, and nerves [1, 2]. Individuals with Type 2 diabetes typically suffer from insulin resistance and relative rather than absolute insulin deficiency. At least initially, and often throughout their lifetimes, these individuals may not require insulin treatment to survive. According to the IDF 2017, 425 million adults have diabetes worldwide, and Mexico ranks 6th among the top ten countries in the number of adults with diabetes.

In Mexico, the use of plants for medicinal purposes has been a common practice since pre-Hispanic times. Among the plants used in the treatment of diabetes,* Bromelia karatas *(L) traditionally known as “piñuela” “chiyol”, “chicipo”, “aguama”, and “cazuela” is a tropical herb that is distributed between 400 and 1500 m elevation. The leaves are elongated and thick and contain sharp teeth along the margins. Inflorescence is sessile at the ground level. Flowering of the plant occurs between May and October. Its distribution in America ranges from Mexico to Ecuador and Brazil; in Mexico, it is mainly found in the tropical zone [3].* B. karatas* is a monocotyledon that produces a variety and diversity of antioxidant metabolites and glucoconjugates such as (6S,9R)-vomifolyl-*β*-D-glucopyranoside, 3,4,5-trimethoxyphenyl-*β*-D-glucopyranoside, 1-O-*β*-D-glucopyranosyl anthranilate, and 3,4-dimethoxyphenyl *β*-D-glucopyranoside [4]. In a previous work, we reported the traditional use of the plant in the treatment of diabetes, and its acute hypoglycemic effect was demonstrated in STZ-NA hyperglycemic rats [5]. That investigation showed that diabetic patients and healers in the municipality of Tlanchinol, Hidalgo, use a decoction of the leaves to control blood glucose levels and confirmed the hypoglycemic effects of aqueous and hydroalcoholic extracts of the plant; these extracts produced the best effects at oral doses of 350 and 300 mg/kg, respectively. The study also reported preliminary experiments to determine the presence of alkaloids, terpenes, and phenolic compounds in the extracts and found that phenolic compounds are the main components. However, to date, the metabolites present in the active extract of* B. karatas* have not been identified.

The aim of the current study was to continue trials of the hypoglycemic effect of administration of a water extract of leaves of* B. karatas* in STZ-NA rats and to evaluate the lipid profiles and glycated hemoglobin levels of the animals after chronic administration of the extract. An additional aim of the study was to characterize the main components of the extract using conventional phytochemical techniques for the separation, isolation, and identification of metabolites.

## 2. Materials and Methods

### 2.1. General Experimental Procedures

NMR spectra including ^1^H, ^13^C, HSQC, HMBC, COSY, NOESY, and TOCSY were recorded in a Varian Inova spectrometer at 400 (^1^H) and 95 MHz (^13^C) or a Bruker DMX500 spectrometer operated at 500 MHz (^1^H) or 125 MHz (^13^C); chemical shifts were recorded as *δ* values. ESI-MS were recorded on a Thermo Scientific LTQ Orbitrap XL hybrid FTMS (Fourier transform mass spectrometer). Data were collected in both positive and negative ionization modes via a liquid chromatographic/autosampler system that consisted of an Agilent HPLC system. Analytical and preparative HPLC analyses were performed in an Agilent 1260 Infinity system equipped with a G1311B Quaternary Pump, a G1367E Autosampler, and a G1315C DAD VL+ and controlled by Agilent ChemStation software. For analytical and semipreparative HPLC, Phenomenex (Luna Omega Polar C_18_, 50 × 2.1 mm id., 1.6 *μ*m) and Macherey-Nagel (Nucleosyl C_18_, 250 × 4.6 mm id., 5 *μ*m and Nucleosyl C_18_, 250 × 10 mm id., 5 *μ*m) columns, respectively, were used. Column chromatography (CC) was conducted on silica gel (70-230 mesh, Merck) or Sephadex LH-20 (Sigma-Aldrich Chemical). Thin-layer chromatography analyses were carried out on silica gel 60 F_254_ plates (Macherey & Nagel) using ceric sulfate (10%) solution in H_2_SO_4_ as the color reagent.

### 2.2. Plant Extracts

The original plant material was collected with the help of the healer “Isabel Escalante” near the town of Tamala, state of Hidalgo, Mexico. The plant species was verified by MC Ramiro Cruz Durán, and a voucher exemplar was deposited at the Herbarium of the “Instituto Mexicano del Seguro Social” (IMSSM 15814). Fresh plant material was collected as needed.

The water extract (WE) was prepared by boiling 20 g of the dry plant material with 500 ml water, followed by filtration and lyophilization to yield 2.119 g of WE. The extract was stored at 4°C for further analysis. The WE and the organic extract (OE) were used for phytochemical identification of the main compounds of* B. karatas*. OE was prepared from 365 g of plant material by consecutive extraction with a mixture of dichloromethane:methanol (1:1). The combined extract was concentrated in a rotary vacuum evaporator (Buchi, Flawil, Switzerland) at 40°C; the resulting concentrate was evaporated to dryness under reduced pressure, yielding 8.42 g of OE.

### 2.3. Experimental Animals

Wistar rats weighing 200-250 g were provided by the Bioterium of the Science School, UNAM; they were acclimated with free access to food and water for at least one week in an air-conditioned room (25°C, 50% humidity) on a 12-h light-dark cycle prior to use in the experiments. All the experiments were conducted in accordance with the principles set forth in the National Institute of Health (NIH) Guidelines for the Care and Use of Laboratory Animals [6]. Hyperglycemia was induced as described by Masiello [7]. In brief, rats that had been fasted overnight were injected intraperitoneally with 150 mg/kg nicotinamide (NA) (Sigma, N3376) 15 min prior to intravenous injection of 65 mg/kg streptozotocin (STZ, Sigma S0130) in citrate buffer. Hyperglycemia was identified by polyuria and polydipsia and by measuring nonfasting plasma glucose levels 48 h after the injection; animals that did not reach glucose levels of 250 mg/dl were rejected.

*Experiment 1. Chronic Hypoglycemic Effect of the Water Extract*. The previously described experimental design [8] was used; in brief, four groups of six rats were used as follows: group I, the normal control (NC), received 1.5 ml of physiological NaCl solution (vehicle); group II, the hyperglycemic control (HC), also received 1.5 ml of physiological NaCl solution; group III was given a standard oral hypoglycemic agent, glibenclamide [5 mg/kg body weight (bw)] in the same vehicle (CG); and group 4 received Bk-WE (218 mg/kg bw) dissolved in 1.5 ml of physiological NaCl solution. The extract or the hypoglycemic agent was orally administered twice daily (in the morning and in the evening) over a period of 42 days. All groups were fed Purina Rodent Laboratory Chow 5001.

Blood was obtained from the tail veins of the animals; the animals were handled according to the procedures described in [6]. Glucose monitoring was performed weekly using glucose test strips and an Accutrend Plus® glucometer. Glycated hemoglobin (HbA1c) was analyzed in a DCA Vantage® Siemens. The lipid profile (HDL, TG and cholesterol) was measured using Cardio Check® and Panels® PTS strips. VLDL was calculated using the following equation: VLDL = 0.2 x TG. HbA1c and lipid profiles were measured on days 0, 14, 28, and 42 after the initiation of treatment. The data were statistically analyzed using the unpaired t-test. The plasma glucose levels are expressed as the mean (S.E.M.).

*Experiment 2. Acute Hypoglycemic Effect of the Isolated Compounds*. To know if the activity is based on the presence of compounds 1-3, or on the presence of any of them in the WE, an acute experiment was performed as previously described [8]. Wistar rats were divided into six groups of four rats each. Group 1, normal control, orally received 1.5 ml of physiological NaCl solution (vehicle), group 2, hyperglycemic control, also received 1.5 ml of physiological NaCl solution, group 3, positive control, received a standard oral hypoglycemic agent [glibenclamide, 5 mg/kg body weight (bw)] in the same vehicle. Groups 4, 5, and 6 were given compounds 1 (72 mg/kg), 2 (1.8 mg/kg), and 3 (3.63 mg/kg) all dissolved in 1.5 ml of physiological NaCl solution. The doses of each compound were calculated according to the radio; plant: yield of the compound.

### 2.4. HPLC Analysis

Elution was conducted at a flow rate of 0.25 mL/min with water containing 0.1% formic acid as solvent A and acetonitrile (MeCN) as solvent B, using a gradient elution of 100:0 (A:B) for 1 min, 95:5 (A:B) at 1.5 min, 85:15 (A:B) at 1.5-12 min, 70:30 (A:B) at 12-20 min, 70:30 (A:B) maintained during 1 min, 60:40 (A:B) at 21-23 min, 20:80 (A:B) at 23-27 min, 10:90 (A:B) at 27-28 min, 10:90 (A:B) maintained during 1 min, and 100:0 (A:B) at 29-31 min. The flow rate was set to 0.25 mL/min, and the temperature of the column was maintained at 35°C. System control, data collection, and data processing were accomplished using OpenLAB LC 1260 chromatography software. Working solutions of samples (WE, OE, fractions and isolated compounds) of* B. karatas* were prepared by dissolving 5.0 mg of the sample in 1 mL of the appropriate solvent (MeOH, MeCN or H_2_O) and injected (2 *μ*L) using an autosampler. For UV detection, the wavelength program was set at an acquisition of *λ* 230, 254, 280, 320, and 365 nm; 320 nm was selected as the optimum wavelength. The equipment, data acquisition, processing, and management of the chromatographic information were controlled with the OpenLab CDS ChemStatiob Edition (2001-2013) software. The separation was carried out using a Phenomenex® Luna Polar C18 (50 x 2.1 mm id., 1.6 *μ*m) reverse phase column; all solvents were purchased from JT Baker as HPLC grade.

### 2.5. Isolation Compounds

WE (1 g) was dissolved in a mixture of MeOH:H_2_O (1:1); from this solution, 241 mg of** 1** spontaneously precipitated. The remainder of the WE was further separated on Sephadex (500 mL MeOH:H_2_O 1:1), yielding 14 primary fractions (WE1-WE14). WE9 (43 mg) was resolved by HPLC (Nucleosil 250 × 10 mm i.d., 5 *µ*m, C18, Macherey-Nagel) using a 15-min gradient of MeCN:MeOH:H_2_O (60:5:35) (2.3 ml/min; 254 and 320 nm), yielding 6 mg of a solid yellow amorphous powder (**2**) with R_T_: 9.53 min. WE13 (46.5 mg) was subjected to preparative TLC (EtOAc:n-hexane:acetone 7:2:1) to yield compound** 3** (12 mg).

The OE (8.42 g) was partitioned by column chromatography (CC) on 336 g of silica gel (70-230 mesh, Merck) using mixtures of n-hexane:CH_2_Cl_2_:MeOH as eluent. Elution was started with 100% n-hexane; the polarity of the eluent was then increased by the addition of increasing amounts of CH_2_Cl_2_ (to 100%) and then MeOH. This yielded 28 primary fractions (OE1-OE28). Fraction OE11 (1.0 g) was subjected to silica gel CC (40 g) using mixtures of n-hexane:CH_2_Cl_2_:MeOH as eluent. Elution was started with 100% n-hexane, and the polarity of the eluent was increased by the gradual addition of CH_2_Cl_2_ (to 100%) and then MeOH. This resulted in 10 subfractions (OE11.1-OE11.10); OE11.10 (40 mg) yielded a mixture of** 4** and** 5**. OE16 (279 mg) was processed with CC on silica gel (11 g) using mixtures of EtOAc:MeOH as eluent, starting with 100% EtOAc and increasing the polarity with MeOH to 100%; this process yielded 9 subfractions (OE16.1-OE16.9). Preparative TLC of OE16.4 (32 mg eluted with n-hexane:EtOAc, 1:9) and OE17 (76 mg eluted with n-hexane:EtOAc; 1:9) resulted in the isolation of** 6** (10 mg) and** 7** (15 mg), respectively. Fraction OE19 (347 mg) was subjected to CC on silica gel (15 g); elution was started with 100% EtOAc, and the polarity of the solvent was increased by the addition of methanol up to 100%; using this process, it was possible to isolate compound** 8** (18 mg). Preparative TLC (EtOAc:MeOH:H_2_O, 8:1.5:0.5) of fraction OE22 (97 mg) resulted in the isolation of** 9** (12 mg). Finally, EO23 (122 mg) and EO24 (434 mg) were dissolved in methanol, and compounds** 2 **(25 mg) and** 1** (10 mg), respectively, were obtained as precipitates.

### 2.6. Identification of the Isolated Compounds in the Chromatographic Profile of WE

Isolated compounds (**1**-**9**) and extracts prepared from* B. karatas* (WE and OE) were injected into the HPLC for identification of the compounds in the chromatographic profiles. Peak assignments were made on the basis of the previously developed structure-diagnostic and supported by examination of the UV spectrum and relative retention time using OpenLAB LC 1260 chromatography software.

## 3. Results

### 3.1. Ethnobotanical Results

In the field, we confirmed the previous finding that the healer “Isabel Escalante” and the diabetic patients of the town of Tamala use a decoction of 30 g of the dry leaves of* B. karatas* boiled in 1 liter of water. The infusion is cooled and drunk over the day as “Agua de Uso”. The common name of the plant in this region is “timbiriche”. Alternatively, the juice of the fruit is used.

### 3.2. Phytochemical Analysis

**Figure 1:**
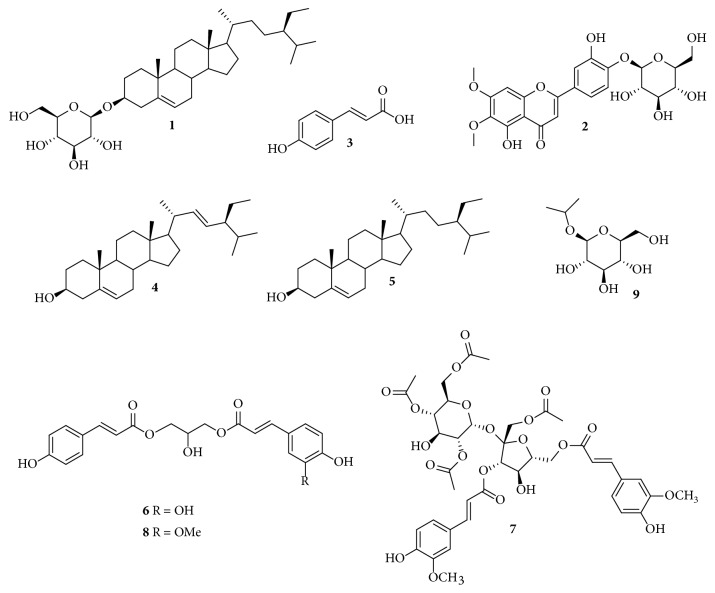
Isolated compounds from* Bromelia karatas*: *β*-Sitosterol-3-O-*β*-D-glucopyranoside, (1), Cirsiliol 4′-O-glucoside (2), *ρ*-Coumaric acid, (3), Stigmasterol (4), *β*-Sitosterol (5), 1-O-Feruloyl-3-O-p-coumaroylglycerol (6), *β*-D-(1-O-Acetyl-3,6-O-trans-diferuloyl)-fructofuranosyl-*α*-D-2′,4′,6′.-O- triacetylglucopyranoside (7), 1-O-p-Coumaroyl-3-O-caffeoylglycerol (8), 2-Propyl-*β*-glucopyranoside (9).

The chromatographic profiles were developed to monitor the major components of WE and OE from* B. karatas* and to make it possible to isolate and identify them by means of various conventional chromatographic and spectroscopic techniques. The analysis led to the isolation of compounds** 1-9**, which were identified based on comparison of their ^1^H and ^13^C-NMR spectral data, including data obtained in 2D experiments (COSY, HSQC, HMBC, NOESY, TOCSY) and their mass spectral data, with those of previously described compounds. The major compounds were identified as a steroidal glycoside, [*β*-sitosterol-3-O-*β*-D-glucopyranoside (**1**)] [9], and a phenolic acid, [*ρ*-coumaric acid, (**3**)] [10]; both compounds were isolated from the traditional extract (WE). The flavonoid [cirsiliol 4′-O-*β*-D-glucopyranoside (**2**)] [11] from WE, along with two steroidal aglycones [stigmasterol (**4**) and *β*-sitosterol (**5**)] [12], two phenylpropanoid glycerols [1-O-feruloyl-3-O-*ρ*-coumaroylglycerol (**6**) and 1-O-*ρ*-coumaroyl-3-O-caffeoylglycerol (**8**)] [13], a phenylpropanoid glycoside [*β*-D-(1-O-acetyl-3,6-O-trans-diferuloyl)fructofuranosyl-*α*-D-2′,4′,6′-O triacetylglucopyranoside (**7**)] [14], and 2-propyl-D-glucopyranoside (**9**) [15] from OE were some of the minor compounds, Figure 1. The complete spectroscopic data of the isolated compounds are presented below.

*β-Sitosterol-3-O-β-D-glucopyranoside, (* ***1***). Colorless powder; FAB-MS m/z 577 [M+H]^+^ for C_35_H_60_O_6_; EI-MS m/z 414 [M-C_6_H_10_O_5_]^+^. ^1^H NMR (400 MHz, Pyridine-*d*_5_) *δ* 5.36 (1H, t,* J* = 2.6 Hz, H-6), 5.07 (1H, d,* J* = 7.8 Hz, H-1′), 4.58 (1H, dd,* J* = 11.8, 2.5 Hz, H-6′), 4.43 (1H, dd,* J* = 11.8, 5.1 Hz, H-6′), 4.30 (2H, m, H-3′ and H-4′), 4.08 (1H, t,* J* = 8.2 Hz, H-2′), 3.99 (2H, m, H-3 and H-5′), 2.75 (1H, ddd,* J* = 13.5, 4.8, 2.2 Hz, H-12), 2.50 (1H, m, H-12), 2.15 (2H, m, H-2), 1.99 (1H, dd,* J* = 12.6, 3.5 Hz, H-4), 1.86 (1H, m, H-4), 1.72 (1H, dtd,* J* = 18.0, 7.9, 6.9, 3.8 Hz, H-1), 1.68 (1H, m, H-25), 1.41 (1H, ddt,* J* = 15.8, 9.8, 4.3 Hz, H-20), 1.36 (1H, m, H-8), 1.10 (1H, m, H-17), 1.02 (1H, m, H-24), 1.00 (3H, d,* J* = 6.5 Hz, H-21), 0.95 (3H, s, H-19), 0.95 (1H, m, H-14), 0.92(3H, d,* J* = 7.3 Hz, H-26) 0.89 (3H, t,* J* = 7.4 Hz, H-29), 0.88 (3H, d,* J* = 6.9 Hz, H-27), 0.67 (3H, s, H-18). ^13^C NMR (100 MHz, Pyridine-*d*_5_) *δ*: 140.91 (C-5), 121.92 (C-6), 102.57 (C-1′), 78.59 (C-3), 78.47 (C-3′), 78.12 (C-5′), 75.30 (C-2′), 71.68 (C-4′), 62.83 (C-6′), 56.84 (C-14), 56.25 (C-17), 50.35 (C-9), 46.05 (C-24), 42.49 (C-13), 39.96 (C-4), 39.34 (C-12), 37.49 (C-1), 36.93 (C-10), 36.40 (C-20), 34.21 (C-22), 32.19 (C-7), 32.06 (C-8), 30.26 (C-2), 29.47 (C-25), 28.56 (C-16), 26.39 (C-23), 24.52 (C-15), 23.40 (C-28), 21.30 (C-11), 20.00 (C-27), 19.43 (C-19), 19.22 (C-26), 19.03 (C-21), 12.17 (C-29), 11.99 (C-18).

*Cirsiliol 4*′*-O-glucoside ( ****2***). Yellow prisms; HR-ESIMS ion at m/z 515.1204 [M+Na]^+^ for C_23_H_24_O_12_. ^1^H NMR (400 MHz, Pyridine-*d*_5_) *δ* 7.96 (1H, d,* J* = 2.2 Hz, H-2′), 7.72 (1H, d,* J* = 8.6 Hz, H-5′), 7.52 (1H, dd,* J* = 8.6, 2.3 Hz, H-6′), 7.03 (1H, s, H-3), 6.73 (1H, s, H-8), 5.80 (1H, d,* J* = 7.7 Hz, H-1′′), 4.61 (1H, dd,* J* = 12.0, 2.1 Hz, H-6_a_′′), 4.44 (1H, dd,* J* = 12.1, 5.3 Hz, H-6_b_′′), 4.38 (1H, t,* J* = 8.9 Hz, H-3′′), 4.34 (1H, d,* J* = 8.9 Hz, H-4′′), 4.26 (1H, t,* J* = 8.9 Hz, H-2′′), 4.18 (1H, ddd,* J* = 9.0, 5.2, 2.1 Hz, H-5′′), 4.02 (3H, s, OCH_3_-6), 3.91 (s, OCH_3_-7). ^13^C NMR (100 MHz, Pyridine-*d*_5_) *δ* 183.55 (C-4), 164.76 (C-2), 159.83 (C-7), 154.02 (C-9), 153.91 (C-5), 150.54 (C-4′), 149.00 (C-3′), 133.57 (C-6), 126.89 (C-1′), 119.7 (C-6′), 118.25 (C-5′), 115.83 (C-2′), 106.87 (C-10), 105.37 (C-3), 103.54 (C-1′′), 91.97 (C-8), 79.68 (C-5′′), 78.91 (C-3′′), 75.18 (C-2′′), 71.57 (C-4′′), 62.69 (C-6′′), 60.96 (6-OCH_3_), 56.84 (7-OCH_3_). 

*ρ-Coumaric Acid, (* ***3***). Colorless amorphous powder; ESI-MS ion at m/z 163 [M-H]^−^ for C_9_H_8_O_3_. ^1^H NMR (400 MHz, CD_3_OD) *δ*: 7.50 (1H, d,* J = *15.7 Hz, H-7), 7.41 (2H, d,* J = *8.6 Hz, H-2, H-6), 6.78 (2H, d,* J= *8.6 Hz, H-3, H-5), 6.33 (1H, d,* J = *15.8 Hz, H-8). ^13^C NMR (100 MHz, CD_3_OD) *δ* 175.20 (C-9), 158.42 (C-4), 144.18 (C-7), 130.54 (C-2, C-6), 128.01 (C-1), 116.66 (C-3, C-5), 115.76 (C-7). 

*Stigmasterol (* ***4***). White powder; ESI-MS ion at m/z 413 [M+H]^+^ for C_29_H_48_O. ^1^H NMR (500 MHz, CDCl_3_) *δ*: 5.35 (1H, m, H-6), 5.15 (1H, dd,* J* = 15.2, 8.0 Hz, H-21), 5.02 (1H, dd,* J* = 15.2, 8.5 Hz, H-20), 3.52 (1H, tdd,* J* = 10.9, 5.5, 4.1 Hz, H-3), 1.01 (3H, s, H-29), 0.92 (3H, d,* J* = 6.6 Hz, H-19), 0.84 (3H, t,* J* = 7.6 Hz, H-24), 0.81 (3H, d,* J* = 6.8 Hz, H-26), 0.80 (3H, d,* J* = 6.8 Hz, H-27), 0.68 (3H, s, H-28). ^13^C NMR (125 MHz, CDCl_3_) *δ* 140.92 (C-5), 138.5 (C-22), 129.4 (C-23), 121.86 (C-6), 71.97 (C-3), 57.03 (C-14), 56.13 (C-17), 51.39 (C-24), 50.31 (C-9), 42.48 (C-4), 42.38 (C-13), 40.64 (C-20), 39.85 (C-12), 37.42 (C-1), 36.67 (C-10), 32.08 (C-7, C-8, C-25), 31.84 (C-2), 29.01 (C-16), 24.46 (C-15), 25.56 (C-28), 21.37 (C-11), 20.36 (C-26), 19.56 (C-27), 18.94 (C-21), 18.94 (C-19), 12.10 (C-29), 12.02 (C-18). 

*β-Sitosterol (* ***5***). White powder; ESI-MS ion at m/z 415 [M+H]^+^ for C_29_H_50_O. ^1^H NMR (500 MHz, CDCl_3_) *δ* 5.35 (1H, d,* J* = 5.3 Hz, H-5), 3.52 (1H, tdd,* J* = 10.9, 5.5, 4.1 Hz, H-3), 1.01 (3H, s, H-29), 0.92 (3H, d,* J* = 6.6 Hz, H-19), 0.83 (3H, d,* J* = 6.8 Hz, H-24), 0.81 (3H, d,* J* = 6.8 Hz, H-26), 0.69 (3H, s, H-28). ^13^C NMR (125 MHz, CDCl_3_) *δ* 140.92 (C-5), 121.86 (C-6), 71.97 (C-3), 57.03 (C-14), 56.13 (C-17), 50.31 (C-9), 46.01 (C-24), 42.48 (C-13), 42.38 (C-4), 39.85 (C-12), 37.42 (C-1), 36.67 (C-10), 36.31 (C-20), 34.12 (C-22), 32.08 (C-2), 31.84 (C-7, C-8), 29.33 (C-25), 28.41 (C-16), 26.26 (C-23), 24.46 (C-15), 23.24 (C-28), 21.37 (C-11), 19.98 (C-26), 19.14 (C-27), 19.13 (C-19), 18.94 (C-21), 12.21 (C-29), 12.02 (C-18).

*1-O-Feruloyl-3-O-p-coumaroylglycerol (* ***6***). Colorless oil; ESI-MS ion at m/z 437.4 [M+Na]^+^ for C_22_H_22_O_8_. ^1^H NMR (400 MHz, CD_3_OD) *δ* 7.66 (2H, d,* J* = 16.0 Hz, H-7′, H-7′′), 7.44 (2H, d,* J* = 8.7 Hz, H-2′, H-6′), 7.18 (1H, d,* J* = 2.0 Hz, H-2′′), 7.07 (1H, dd,* J* = 8.3, 2.1 Hz, H-6′′), 6.80 (2H, d,* J* = 8.2 Hz, H-3′, H-5′), 6.79 (1H, d,* J* = 8.6 Hz, H-5′′), 6.39 (1H, d,* J* = 14.2 Hz, H-8′′), 6.35 (1H, d,* J* = 14.3 Hz, H-8′), 4.28 (4H, dd,* J* = 5.3, 1.9 Hz, H-1, H-3), 4.16 (1H, m, H-2), 3.87 (3H, s, OCH_3_-3′′). ^13^C NMR (100 MHz, CD_3_OD) *δ* 169.01 (C-9′′), 168.99 (C-9′), 161.34 (C-4′), 150.71 (C-3′′), 149.38 (C-4′′), 147.25 (C-7′′), 146.97 (C-7′), 131.21 (C-2′, C-6′), 127.66 (C-1′′), 127.11 (C-1′), 124.19 (C-6′′), 116.84 (C-3′, C-5′), 116.48 (C-5′′), 115.10 (C-8′′), 114.80 (C-8′), 111.75 (C-2′′), 68.61 (C-2), 66.39 (C-3), 66.37 (C-1), 56.44 (OCH_3_-3′′).

*β-D-(1-O-Acetyl-3,6-O-trans-diferuloyl)-fructofuranosyl-α-D-2*′*,4*′*,6*′*.-O- triacetylglucopyranoside ( ****7***). Colorless amorphous solid, ESI-MS ion at m/z 885.2 [M+Na]^+^ for C_40_H_46_O_21_. ^1^H NMR (500 MHz, CD_3_OD) *δ* 7.72 (1H, d,* J =* 15.9 Hz, H-7′′), 7.68 (1H, d,* J =* 15.9 Hz, H-7′′′), 7.28 (2H, d,* J =* 2.0 Hz, H-2′′), 7.22 (2H, d,* J =* 2.0 Hz, H-2′′′), 7.13 (1H, dd,* J = *8.5, 2.01 Hz, H-6′′), 7.11 (1H, dd,* J = *8.5, 1.9 Hz, H-6′′′), 6.83 (2H, d,* J =* 8.2 Hz, H-5′′, H-5′′′), 6.46 (1H, d,* J =* 14.42 Hz, H-8′′), 6.43 (1H, d,* J =* 14.4 Hz, H-8′′′), 5.70 (1H, d,* J =* 3.8 Hz, H-1′), 5.39 (1H, d,* J =* 7.7 Hz, H-3), 4.80 (1H, d,* J =* 10.2 Hz, H-4′), 4.68 (1H, dd*, J =* 10.2, 3.8 Hz, H-2′), 4.50 (1H, dd,* J = *12.1, 3.27 Hz, H-6_a_) 4.45 (1H, m, H-6_b_), 4.46 (1H, t,* J = *7.9 Hz, H-4), 4.26, (d,* J = *11.5 Hz, H-1_a_), 4.24 (1H, m, H-5′), 4.21 (d br,* J =* 11.4 Hz, H-6′_a_), 4.10 (1H, dd,* J = *11.6, 3.5 Hz, H-6′_b_) 4.09 (1H, d,* J =* 11.5 Hz, H-1_b_), 3.91 (3H, s, OCH_3_-3′′′), 3.91 (3H, s, OCH_3_-3′′), 3.88 (1H, d,* J =* 7.6 Hz, H-3′), 2.13 (3H, s, OAc-1), 2.10 (3H, s, OAc-2′), 2.03 (3H, s, OAc-6′), 1.87 (3H, s, OAc-4′). ^13^C NMR (125 MHz, CD_3_OD) *δ* 172.60 (OAc-6′), 172.14 (OAc-2′), 172.02 (OAc-1), 171.72 (OAc-4′), 168.77 (C-9′′′), 167.98 (C-9′′), 151.12 (C-4′′), 150.76 (C-4′′′), 149.51 (C-3′′), 149.43 (C-3′′′), 148.23 (C-7′′), 147.25 (C-7′′′), 127.74 (C-1′′), 127.50 (C-1′′′), 124.62 (C-6′′), 124.31 (C-6′′′), 116.57 (C-5′′), 116.52 (C-5′′′), 115.15 (C-8′′′), 114.30 (C-8′′), 111.83 (C-2′′), 111.68 (C-2′′′), 103.91 (C-2), 90.46 (C-1′), 81.52 (C-5), 79.55 (C-3), 73.95 (C-2′), 73.79 (C-4), 72.25 (C-4′), 70.06 (C-5), 69.82 (C-3′), 66.61 (C-1), 64.53 (C-6), 64.09 (C-6′), 56.56 (OCH_3_-3′′, OCH_3_-3′′′), 20.92 (OAc-CH_3_-2′), 20.79 (OAc-CH_3_-1), 20.68 (OAc-CH_3_-6′), 20.62 (OAc-CH_3_-4′). Assignments of the acetate and feruloyl units at positions 1, 2′, 4′, 6′ (for acetyl) and 3, 6 (for feruloyl), respectively, in the sucrose molecule were corroborated by the HMBC two-dimensional spectrum; this showed a correlation between methine and methylene protons at 1, 2′, 4′ and 6′ of the sucrose with methyl protons and the carbonyl carbon of the acetyl groups (^1^H/^13^C/^1^H: 4.26, 4.09/172.02/2.13 for 1; 4.68/172.14/2.10 for 2′; 4.80/171.72/1.87 for 4′; 4.21, 4.10/172.60/2.03 for 6′) and between methine and methylene protons at 3 and 6 of the fructose with the protons at 8′′ and 8′′′ of the feruloyl moiety and the carbonyl carbons at 9′′ and 9′′′ (^1^H/^13^C/^1^H: 6.46/167.98/5.39 for C-3 feruloyl; 6.43/168.77/4.50, 4.45 for C-3 feruloyl). 

*1-O-p-Coumaroyl-3-O-caffeoylglycerol (* ***8***). Colorless oil; ESI-MS ion at m/z 399.8 [M-H]^−^ for C_21_H_20_O_8_.^1^H NMR (400 MHz, CD_3_OD) *δ* 7.66 (1H, d,* J =* 16.0 Hz, H-7′), 7.60 (1H, d,* J =* 15.9 Hz, H-7′′), 7.44 (2H, dd,* J =* 8.9, 2.3 Hz, H-2′, H-6′), 7.05 (1H, d,* J =* 2.1 Hz, H-2′′), 6.94 (1H, dd,* J = *8.3, 2.0 Hz, H-6′′), 6.79 (2H, dd*, J =* 9.1, 2.5 Hz, H3′, H-5′), 6.77 (1H, d,* J =* 8.4 Hz, H-5′′), 6.35 (1H, d,* J = *15.9 Hz, H-8′), 6.30 (1H, d,* J =* 15.9 Hz, H-8′′), 4.27 (4H, d,* J =* 5.4 Hz, H-1, H-3), 4.16 (1H, q,* J =* 5.2 Hz, H-2). ^13^C NMR (100 MHz, CD_3_OD) d 169.01 (C-9′, C-9′′), 161.31 (C-4′), 149.65 (C-3′′), 147.36 (C-7′′), 146.97 (C-7′), 146.81 (C-4′′), 131.22 (C-2′, C-6′), 127.70 (C-1′′), 127.13 (C-1′), 123.02 (C-6′′), 116.83 (C-3′, C-5′), 116.51 (C-5′′), 115.20 (C-2′′), 114.82 (C-8′), 114.76 (C-8′′), 68.65 (C-2), 66.35 (C-1, C-3). 

*2-Propyl-β-glucopyranoside (* ***9***). Colorless amorphous solid, ESI-MS ion at m/z 245.2 [M+Na]^+^ for C_9_H_18_O_6_. ^1^H NMR (400 MHz, CD_3_OD) *δ* 4.34 (1H, d,* J =* 7.8 Hz, H-1), 4.04 (1H, hept,* J =* 6.1 Hz, H-2′), 3.85 (1H, dd,* J = *11.9, 2.0 Hz, H-6_b_), 3.66 (1H, dd,* J = *11.9, 5.4 Hz, H-6_a_), 3.35 (1H, m, H-3), 3.27 (1H, m, H-4), 3.25 (1H, m, H-5), 3.14 (1H, dd,* J = *9.1, 7.8 Hz, H-2), 1.23 (3H, d,* J =* 6.2 Hz, CH_3_-1′), 1.19 (3H, d,* J = *6.1 Hz, CH_3_-3′). ^13^C NMR (100 MHz, CD_3_OD) *δ* 102.55 (C-1), 78.11 (C-3), 77.86 (C-5), 75.14 (C-2), 72.58 (C-2′), 71.70 (C-4), 62.79 (C-6), 23.81 (C-1′), 22.04 (C-3′).

### 3.3. Chromatographic Profiles

**Figure 2:**
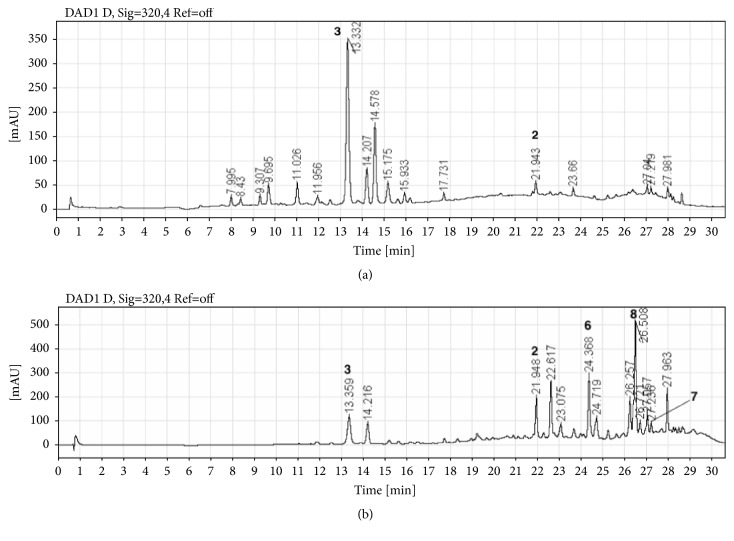
HPLC-DAD profiles of the WE and the OE. (a) Water extract; (b) organic extract.

In the HPLC profiles of the WE and the OE of* B. karatas* (Figure 1), the majority of components showed maximum absorption between 280 and 320 nm. The two chromatograms were qualitatively different; however, the main components of WE [T_R_: 13.3 (**3**), 14.2 and 21.9 (**2**) min] were also observed in the OE [T_R_: 13.3 (**3**), 14.2 and 21.9 (**2**) min] but in different proportion. Compounds** 4**,** 5,** and** 9** were not observed in any of the chromatographic profiles, Figure 2.

Compound** 1** was not observed as a major compound in the profile of the WE (Figure 2(a)) because it precipitated during the process of sample preparation before the sample was injected into the HPLC equipment; its remnant was observed at 254 nm at a retention time of 30.65 min (data not shown).

### 3.4. Hypoglycemic Effect of the Water Extract in a Chronic Trial for 42 Days

After the induction of experimental hyperglycemia, the HC group presented higher glucose values than the NC group throughout the 42-day duration of the study, while in the GB group, a hypoglycemic effect was observed from days 7 to 42; this effect was statistically significant compared with the HG group and with the time 0 values of the GB group. A similar hypoglycemic effect beginning at day 7 and continuing until day 42 was observed after the oral administration of BK-WE, confirming that both the plant extract and the drug glibenclamide exert a chronic hypoglycemic effect (see Table 1).

The levels of glycated hemoglobin were elevated in the HC group compared to the NC, CG, and BK-WE groups. Both glibenclamide and the extract controlled the increase in the values of HB1Ac, but the effect was not statistically significant compared to the HG or to the animals' own time 0 values. The cholesterol values were similar in all the groups (data not shown), and vLDL levels were not modified by the control drug or by the extract (see Table 2).

### 3.5. Hypoglycemic Effect of the Isolated Compounds

After the induction of experimental hyperglycemia, the hyperglycaemic group (HC) presented higher glucose values than the normoglycemic group (NC). Through the 180 minutes, both groups present a stable glycemia, the NC around 100 mg/dl and the HC around 190 mg/dl, with no statistical difference between their own time 0. However, HC presents higher glucose levels compared to NC. When the hypoglycemic agent glibenclamide (5 mg/kg) was administrated (GC) a statistically significant hypoglycemic effect from 60 to 180 min compared against the HC and their own time 0 could be observed. The compound (1) *β*-Sitosterol-3-O-*β*-D-glucopyranoside exerts a hypoglycemic effect, but it is only statistically significant at 180 min. The compound (2) Cirsiliol 4′-O-glucoside exerts a statistically significant hypoglycemic effect from 60 to 180 min, like glibenclamide. The compound (3) *ρ*-Coumaric acid also produces a hypoglycemic effect since 120 min, but it is only statistically significant at time 180 min (see Table 3).

## 4. Discussion

The results of the present work support the traditional use of* B. karatas* in the treatment of type 2 diabetes. The extract tested here, which is similar to the traditionally used infusion, possesses a chronic hypoglycemic effect, and the observed effect was sustained throughout a 42-day period. The plant extract was also able to control the elevation in glycated hemoglobin with no effect on cholesterol or vLDL levels.

The chromatographic profiles and the absorption spectra of the extracts of* B. karatas* indicated the presence of phenolic compounds and flavonoids. The organic extract was used to increase the chemical profile of the plant, making it possible to isolate several phenolic compounds, including glycosides and glycerides of phenylpropanoids, flavonoids, and phytosterols.** 1**,** 4,** and** 5** are the major phytosterols in higher plants;** 2** is part of the structural group of polymethoxylated flavones, which are distributed mainly in the Rutaceae and have been shown to have a broad spectrum of biological activity that includes anti-inflammatory, anticancer, and antiatherogenic properties [16];** 3, 6, **and** 8 **have been previously described in the family Bromeliaceae [17];** 7** was isolated for the first time from* Sparganium stoloniferum* [14]; and** 9** is not commonly isolated from natural products. Sharma et al. (1989) [18] isolated the isopropyl-*α*-D-glucopyranoside from the coral* Sclerophytum capitalis*; they assumed that this compound is not an artifact produced during the investigation because, similar to us, they did not use propanol in the extraction and purification process. It should be noted that** 1**-**9** have not been reported previously for* B. karatas*.** 1** and** 3** were considered the major compounds in the infusion (WE). Several studies show that saponins regulate blood glucose levels and prevent diabetic complications due to their antioxidant activity [19]; sitosterol-3-O-*β*-D-glucopyranoside (**1**) was shown to have hypoglycemic and antihyperglycemic effects in STZ-NA rats treated with doses of 0.25 and 0.50 mg/kg for 21 days using glibenclamide as a positive control and to improve biochemical and hematological parameters such as total cholesterol, triglycerides, high-density lipoprotein (HDL), low-density lipoprotein (LDL), blood urea nitrogen, creatinine, red blood cells, platelets, and white blood cells, [20]. On the other hand, Amalam et al. (2016) [21] demonstrated the “antidiabetic” potential of *ρ*-coumaric acid (**3**) by showing that it exerts a protective role in pancreatic b-cells of diabetic rats by reducing ROS-induced oxidative stress and improving antioxidant status and by providing evidence for the participation of GLUT-2 in controlling glucose homeostasis. A more recent trial showed the powerful antihyperglycemic and antihyperlipidemic efficacy of *ρ*-coumaric acid in STZ-NA rats treated orally with 40 mg/kg body mass for six weeks; this process may be mediated via modulation of TNF-*α* and adipocytokine secretion as well as by upregulation of PPAR*γ* mRNA expression [22]. The effect of the WE from* Bromelia karatas* in this test was similar to that of glibenclamide; consequently, we were able to confirm that** 1** and** 3** are the active principles of the plant.

The previously reported hypoglycemic effect of compounds** 1** and** 3** is here supported by our own data, for *β*-Sitosterol-3-O-*β*-D-glucopyranoside we observe nearly 80% of glucose reduction after 180 min, a similar result was previously described in a chronic experiment [20], and also the previous observation about the hypoglycemic effect of *ρ*-Coumaric acid observed by [21] was corroborated here. For the first time, the hypoglycemic effect of Cirsiliol 4′-O-glucoside is reported, which exerts better effect with a 30% reduction in blood sugar levels after 180 min.

This work supports the traditional use of the plant to treat type 2 diabetes and describes the compound Cirsiliol 4′-O-glucoside as a novel hypoglycemic agent; further investigations are needed to establish the hypoglycemic mechanism of the plant and the compound Cirsiliol 4′-O-glucoside.

## Figures and Tables

**Table 1:** Chronic hypoglycemic effect of *Bromelia karatas* on STZ-NA induced diabetic rats.

Glucose Groups	(mg/dl)	(mg/dl)	(mg/dl)	(mg/dl)	(mg/dl)	(mg/dl)	(mg/dl)
	T0	T7	T14	T21	T28	T35	T42
NC	124 ± 3.2	129 ± 1.7	124 ± 2.4	127 ± 1.1	125 ± 4.3	118 ± 6.4	131 ± 2.7

HC	174 ± 10.6^1^	171 ± 1.5^1^	172 ± 9.7^1^	153 ± 3.8^1^	168 ± 7.3^1^	162 ± 3.8^1^	169 ± 8.7^1^

CG	180 ± 2.5^1^	128 ± 4.9 ^a,1^	147 ± 9.4 ^a,1^	133 ± 6.2 ^a,1^	150 ± 9 ^a,1^	134 ± 11.4 ^a,1^	152 ± 10.8 ^a,1^
5 mg/kg							

Bk-WE	186 ± 3.1^1^	138 ± 3.3 ^a,1^	146 ± 2.8 ^a,1^	138 ± 3.1 ^a,1^	141 ± 5.1 ^a,1^	132.8 ± 4.2 ^a,1^	140.2 ± 7.8 ^a,1^
218 mg/Kg							

The values represent the mean ± SEM. Superscripted numbers in the same column indicate statistically significant differences compared with the respective control group. Superscripted letters in the same row indicate statistically significant differences compared with time 0. a,1 (p < 0.05).

**Table 2:** Chronic hypoglycemic effects of *Bromelia karatas* on STZ-NA induced diabetic rats.

	*T0*		*T14*		*T28*		*T42*	
Groups	*HbA1c*	*vLDL*	*HbA1c*	*vLDL*	*HbA1c*	*vLDL*	*HbA1c*	*vLDL*
	*(%)*	*(mg/dl)*	*(%)*	*(mg/dl)*	*(%)*	*(mg/dl)*	*(%)*	*(mg/dl)*
NC	3.6 ± 0.1	14.2 ± .8	3.6 ± 0.1	15.8 ± 3	3.5 ± 0.1	12.6 ± .8	3.6 ± 0.1	12.2 ± 2

HC	3.7 ± 0.1	10.4 ± 1	4.1 ± .2	15 ± 2.7	4.2 ± 0.1 ^1,a^	23.8 ± 3^1,a^	4.3 ± 0.1^1,a^	22.6 ± 5 ^a^

CG	3.8 ± 0.2	13.8 ± 2	4.2 ± 0.2 ^1,a^	17.8 ± 3	3.9 ± 0.2	20 ± 3	3.9 ± 0.2	23 ± 3.3 ^a^
5 mg/kg								

Bk-WE	3.6 ± 0.1	20 ± 2^1^	4.1 ± 0.2	21 ± 4.6	4.0 ± 0.1	17.2 ± 3	4.1 ± 0.1	25 ± 6
218 mg/Kg								

The values represent the mean ± SEM. Superscripted letters in the same row indicate statistically significant differences compared with time 0. Superscripted numbers in the same column indicate statistically significant differences compared with the respective control group. a,1 (p < 0.0). VLDL was calculated using the following VLDL = 0.2 x TG.

**Table 3:** Acute hypoglycemic effect of the isolated compounds.

	Glucose levels in the maltose curve [mg/dl]			
Group/Time (min.)	T0	T60	T120	T180
Normoglycemic control	106 ± 5 ^b^	114 ± 9^b^	110 ± 7^b^	105 ± 5^b^

Hyperglycaemic control	196 ± 7	191 ± 7	187 ± 3	194 ± 6

Hyperglycemic + glibenclamide	192 ± 8	118 ± 10^a,b^	107 ± 7^a,b^	106 ± 9^a,b^
5 mg/kg				

*β-Sitosterol-3-O-β-D-glucopyranoside. *72 mg/kg	186 ± 6	203 ± 5	172 ± 2	156 ± 7^b^

*Cirsiliol 4*′*-O-glucoside*	194 ± 5	179 ± 8^a^	160 ± 10^a,b^	133 ± 14^a,b^
1.8 mg/kg				

*ρ*-Coumaric acid	187 ± 8	198 ± 7	180 ± 7^a^	166 ± 9^a^
3.63 mg/kg				

The values represent the mean ± SEM. In the same row: a indicates statistically significant differences compared with time 0. In the same column: b indicates statistically significant differences compared with the diabetic control group; p < 0.05, n = 3.

## Data Availability

The data used to support the findings of this study are available from the corresponding author upon request.
