# Corrigendum to “Phytochemical Composition and Chronic Hypoglycemic Effect of *Bromelia karatas* on STZ-NA-Induced Diabetic Rats”

**DOI:** 10.1155/2021/9758317

**Published:** 2021-06-09

**Authors:** Sonia M. Escandón-Rivera, Adolfo Andrade-Cetto, Gabriela Sánchez-Villaseñor

**Affiliations:** Laboratorio de Etnofarmacología, Facultad de Ciencias, Universidad Nacional Autónoma de México, 4510 Ciudad de México, Mexico

In the article titled “Phytochemical Composition and Chronic Hypoglycemic Effect of *Bromelia karatas* on STZ-NA-Induced Diabetic Rats” [1], the authors wish to additionally acknowledge DGAPA-UNAM for the postdoctoral scholarship provided to Sonia M. Escandón-Rivera. The revised acknowledgments statement is as follows:
